# A Heterozygous Novel Mutation in *TFAP2A* Gene Causes Atypical Branchio-Oculo-Facial Syndrome With Isolated Coloboma of Choroid: A Case Report

**DOI:** 10.3389/fped.2020.00380

**Published:** 2020-07-17

**Authors:** Jie Min, Bing Mao, Yong Wang, Xuelian He, Shuyang Gao, Hairong Wang

**Affiliations:** ^1^Department of Obstetrics and Gynecology, Wuhan Union Hospital, Tongji Medical College, Huazhong University of Science and Technology, Wuhan, China; ^2^Department of Neurology, Wuhan Children's Hospital, Tongji Medical College, Huazhong University of Science and Technology, Wuhan, China; ^3^Wuhan Aier Eye Hospital, Aier School of Ophthalmology, Central South University, Wuhan, China; ^4^Department of Obstetrics and Gynecology, Wuhan Medical and Health Center for Women and Children, Wuhan, China; ^5^BGI Genomics, BGI-Shenzhen, Shenzhen, China; ^6^BGI-Wuhan Clinical Laboratories, BGI-Shenzhen, Wuhan, China

**Keywords:** branchio-oculo-facial syndrome, coloboma of choroid, *TFAP2A* gene, next-generation sequencing, genotype-phenotype

## Abstract

**Background:** Branchio-oculo-facial syndrome (BOFS) is a rare congenital developmental disorder with highly variable clinical phenotypes in autosomal dominant inheritance. The aim of this study is to identify disease-causing mutations in a Chinese family with predominant coloboma of choroid.

**Case report:** We described a family (a mother and her daughter) with unclear clinical diagnosis. The mother (proband) presented with bilateral coloboma of choroid, whereas her daughter had a relatively severe phenotype and presented with larger bilateral choroid coloboma and high-vaulted arch. We applied the next generation sequencing (NGS) panel and analyzed 776 genes related to inherited ocular disorders on the proband. Four candidate heterozygous variants in four genes, respectively, were detected in the proband. Validation of these variants were subsequently performed in the family using Sanger sequencing. Among these variants, a novel nonsense mutation c.912C>A, p.(Cys304^*^) (NM_001042425.2) which in exon 6 of the conserved helix-span-helix domain in *TFAP2A* results in a premature termination codon. It may trigger nonsense-mediated mRNA decay (NMD). Both the affected mother and daughter had this variant, whereas it was absent in the asymptomatic father. Together with the silicon tools and clinical features, we concluded that the variant c.912C>A, p.(Cys304^*^), was the second reported nonsense mutation in *TFAP2A* gene, which was the disease-causing mutation of the family.

**Conclusion:** There are many hereditary diseases accompanied by ocular anomalies. For instance, BOFS, patients with atypical features are always at risk of being under-diagnosed. NGS is a powerful method to identify the genetic cause and improve genetic counseling for less clarified hereditary ocular diseases.

## Introduction

Branchio-oculo-facial syndrome (BOFS; OMIM 113620) is a rare congenital developmental disorder with highly variable clinical features in autosomal dominant inheritance. It is characterized by three main features: branchial skin defects, ophthalmic malformations, and craniofacial anomalies (1). Branchial characteristics include atrophic, hemangiomatous cervical, or supra-auricular. Ocular features are highly variable expressed including microphthalmia, anophthalmia, coloboma of choroid and/or iris, cataract, ptosis, and strabismus. Craniofacial defects include cleft lip (CL) with or without cleft palate (CP), broad nasal bridge, inner ear malformation, hearing loss (conductive and/or sensorineural) and a high forehead (2, 3). Additional symptoms observed include ectodermal anomalies of the hair, teeth, and nails, prenatal and postnatal growth restriction, congenital heart defects, renal malformations, temporal bone anomalies, whereas developmental delay and/or psychiatric are not common (4, 5). The cases of BOFS are distinctive rare, the prevalence of the BOFS is unknown.

*TFAP2A* (6p34.3) is the gene reported to date responsible for the development of BOFS. It encodes transcription factor AP-2 alpha (AP-2α). AP-2α belongs to a member of the AP-2 family of transcription factors (3). AP-2α is a 52-kD retinoic acid-inducible transcription factor that binds to the consensus sequence 5′-GCCNNNGGC-3′ as a dimer and forms homodimers or heterodimers with other similar family members. AP-2α plays the role of gatekeepers balancing the proliferation and differentiation of embryogenesis (6). It plays a role with other signals to regulate the morphogenesis of the eye, face, body wall, limb, neural tube, and kidney (3, 7). BOFS shows broad phenotypic features ranging from mild to severe forms. No more than 150 cases have been reported with a well-illustrated clinical or molecular diagnosis until now (8). Most individuals with BOFS can be diagnosed correspondence with all three or two features as well as the first-degree affected relative or ectopic thymus (3). Nevertheless, in some cases, the patients showed atypical phenotypic features (9–11).

For the individuals who not meet clinical diagnostic criteria, the molecular genetic testing approaches such as the multigene panel or even exome sequencing could assist clinical diagnosis. The combination of genetic analysis and clinical features is a method for accurate diagnosis, especially helpful for the genetic counseling and prenatal diagnosis.

In the present study, we described a mother and her daughter with an atypical BOFS phenotype that had a heterozygous mutation of the *TFAP2A* gene which was identified by NGS.

## Case Presentation

### Clinical Examination

**Figure 1:**
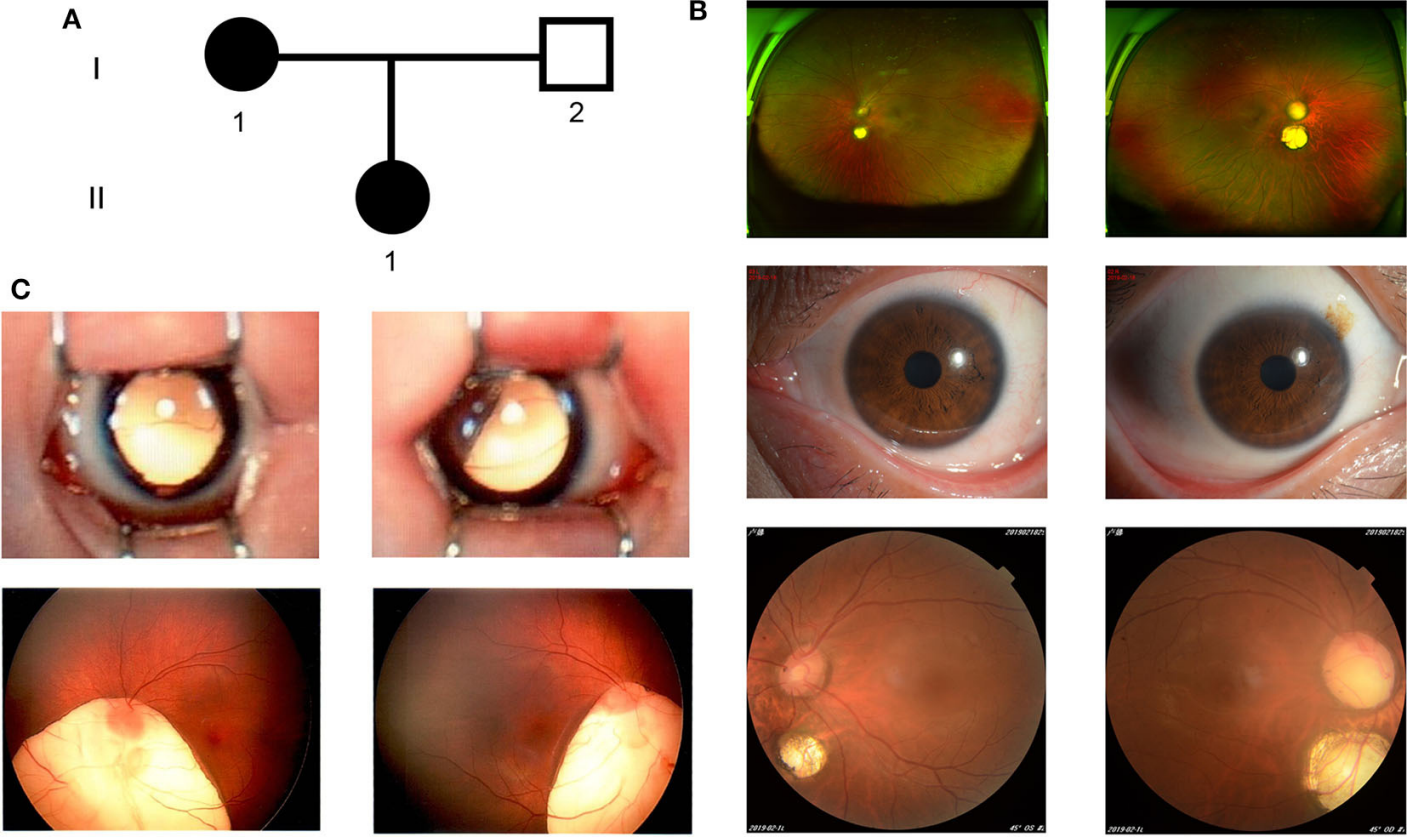
The clinical features of the family. **(A)** The family pedigree. **(B)** Color fundus photographs (Panoramic, anterior, and posterior) of the I-1. **(C)** Color fundus photographs (Panoramic and posterior) of the II-1. left column: left eye; right column: right eye.

The proband (I-1) (Figure 1A) was a 38-year-old Chinese woman, she presented with bilateral choroid colobomas. She accepted the careful ophthalmologic examination at Wuhan Aier Eye Hospital. Her binocular visual acuity was 1.0, bilateral intraocular pressure was 17 mmHg. Fundus examination revealed choroidal coloboma in both eyes. One choroidal coloboma which covered an area of 1 papillary diameter (PD), was located in 1 PD underneath the optic disc of the left eye. The other one which covered an area of 2 PD, was located in 0.5 PD underneath the optic disc of the right eye (Figure 1B). She did not have any other defects after a detailed physical inspection. Her daughter (II-1) (Figure 1A) was 6 years old and had a relatively severe phenotype presented with larger bilateral choroidal coloboma, plus a high-vaulted arch. One choroidal coloboma existed around the 1/3 inferior quadrant underneath the left eye's optic disc, the other one existed around the 1/4 inferior quadrant underneath the right eye's optic disc (Figure 1C). The other physical examinations were unremarkable. The proband's husband (I-2) (Figure 1A) had no ocular and other developmental anomalies. I-1 and I-2 were non-consanguineous Chinese couple.

### Targeted Next-Generation Sequencing (Targeted NGS)

In order to identify the pathogenic mutation underlying the eye defects in this family, we applied the NGS on the proband with the 4 K captured chip which was described in previously published literature (12). The panel consisted of 4,689 nuclear genes of Mendelian diseases. We focused on the 776 genes related to inherited ocular disorders for the variant analysis (Table S1). The genomic DNA was isolated from the 5 ml blood sample with QIAamp DNA Blood Midi Kit (Qiagen, Hilden, Germany). The genomic DNA was fragmented into 200–250 bp by Covaris LE220(Massachusetts, USA). BGISEQ-500 library was enriched by array hybridization, elution, and amplification. The prepared library was sequenced using BGISEQ-500 platform (BGI, Shenzhen, China) which was based on the DNA NanoBalls (DNBs) technology according to the previous paper (13). SOAPnuke was used to generate the “clean reads” (90 bp in length) from the raw sequencing reads. The Burroughs Wheeler Aligner (BWA) was applied to align the clean reads to the GRCh37p10(hg19). SOAPsnp and Samtools software were used to call SNVs and Indels. All the variants were filtered against dbSNP (https://www.ncbi.nlm.nih.gov/SNP), HapMap (https://www.internationalgenome.org/category/hapmap), 1 k Genomes (www.internationalgenome.org), ExAC (https://gnomad.broadinstitute.org/) and 100 Chinese healthy adults database with allele frequency <0.05. The pathogenic impact of the variants was predicted using three algorithms: PolyPhen-2 (http://genetics.bwh.harvard.edu/pph2/), SIFT (http://sift.jcvi.org/) and MutationTaster (http://www.mutationtaster.org).

The quality control (QC) of sequence reads generated targeted NGS has been performed following the previous literature (14). The data of QC were summarized in Table S2. The size of the target region (776 genes of inherited ocular disorders) was 4.05 Mb, and the average sequencing depth was 154.05-fold. 99.99% of targeted bases were covered and 99.74% reads had an average depth which was at least 10-fold. Four novel heterozygous variants were predicted as candidate variants in the proband for further analysis (Table S3). They did not exist in any above databases. The first variant was a nonsense mutation c.912C>A, p.(Cys304^*^) (NM_001042425.2) in exon 6 of *TFAP2A* gene, and it was supported by 112 out of 240 reads. It caused a premature termination codon (PTC) and might trigger nonsense-mediated mRNA decay (NMD). It is an ocular coloboma-associated gene. The second was a small insertion (c.1130+2dup(NM_003816.2) in intron 11 of *ADAM9* gene, and it was supported by 55 out of 110 reads. This mutation was predicted to disturb 5′- splice sites. Mutations in *ADAM9* gene underline autosomal recessive Cone-rod dystrophy 9 (OMIM 612775), characterized by retinal pigment deposits visible on fundus examination. The third one was a missense mutation c.43G>A, p.(Gly15Ser) (NM_015645.5) in exon 18 of *C1QTNF5* gene, it was supported by 24 out of 47 reads. This variant was “tolerable” which was predicted by SIFT and “polymorphism” by Mutation Taster. Defects in *C1QTNF5* gene are a cause of autosomal dominant late-onset retinal degeneration (OMIM 605670), characterized by night blindness in the fifth or sixth decade of life. The last was a missense mutation c.2791G>A p.(Glu931Lys) (NM_001142800) in *EYS* gene, it was supported by 46 out of 97 reads. This variant was annotated “damaging” by SIFT, “benign” by PolyPhen-2, and “polymorphism” by Mutation Taster (Table S1). Mutations in *EYS* gene are responsible for patients with autosomal recessive retinitis pigmentosa 25 (OMIM 602772), characterized by progressive retinal degeneration. The nonsense variant c.912C>A, p.(Cys304^*^) in *TFAP2A* was assessed as likely pathogenic mutation, the other three variants were assessed as uncertain significance (VUS) based on the ACMG 2015 guidelines.

### Sanger Sequencing

**Figure 2:**
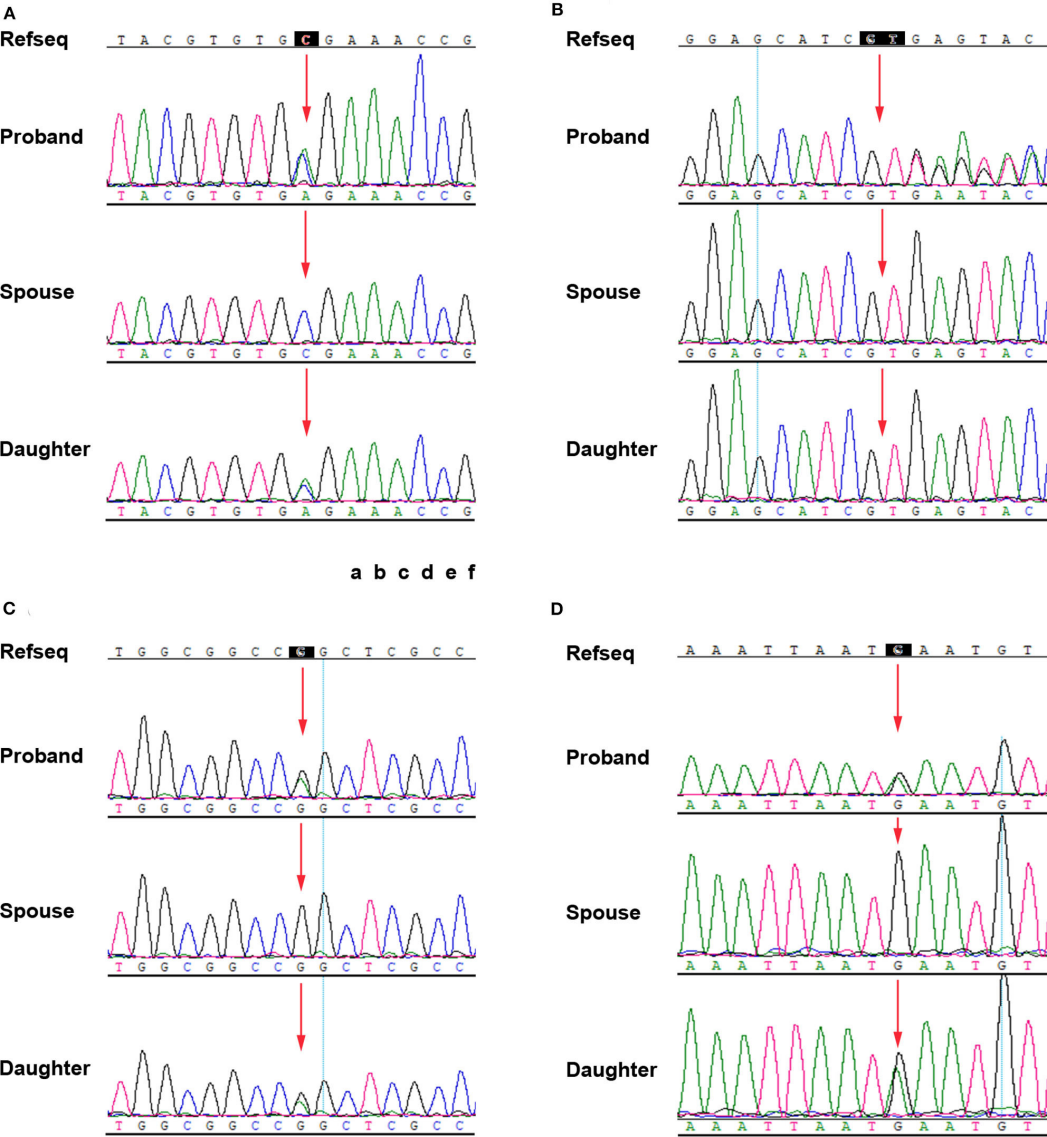
Confirmation of the four mutations by Sanger sequencing. **(A)** *TFAP2A* NM_001042425.2: c.912C>A p.(Cys304*). **(B)** *ADAM9* NM_003816.2: c.1130+2dup. **(C)** *C1QTNF5* NM_015645.5: c.43G>A p.(Gly15Ser). **(D)** *EYS* NM_001142800: c.2791G>A p.(Glu931Lys).

Sanger sequencing for the identified variants was performed on the I-1, I-2, and II-1. The primer pairs were listed in Table S4. The proband's asymptomatic parents were not available for co-segregation analysis. The results revealed that I-1and II-1 had the heterozygous variants, c.912C>A, p.(Cys304^*^) in *TFAP2A*, c.43G>A, p.(Gly15Ser) in *C1QTNF5*, c.2791G>A, p.(Glu931Lys) in *EYS* gene, I-2 did not have mutations at these sites. I-2 and II-1 had the heterozygous variant c.1130+2dup in *ADAM9*. I-1 is normal at this site (Figure 2). By combining the clinical characteristics, genetic mode, and silicon analysis results, we propose that mutation c.912C>A in *TFAP2A* was likely causative mutation for the features of I-1 and II-1.

## Discussion and Conclusion

I-1 and II-1 in our report both had bilateral choroidal coloboma, we analyzed the 776 genes related to inherited ocular disorders with targeted NGS approach. We especially centered on the genes which disturb the function of the pathogenesis of coloboma. Through systematic analysis, we finally took the variant in *TFAP2A* into consideration.

**Figure 3:**
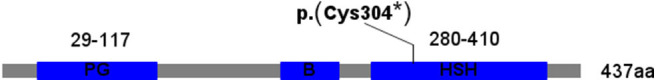
The protein structure of the TFAP2A. PG, proline and glutamine rich domain; B, basic DNA binding domain; HSH, helis span helix domain.

The *TFAP2A* gene maps to chromosome 6p24 and consists of seven exons encoded transcription factor AP-2α with 437 amino acids (NM_001042425). AP-2α contains three conserved domains: a proline and glutamine rich (PG) domain in the N-terminal region, which is responsible for transcriptional activation; a central basic DNA binding (B) domain; and a highly conserved helix-span-helix (HSH) domain in the C-terminal region, which interferes with dimerization and site-specific DNA binding (Figure 3). *TFAP2A* mutations or deletions are known to the genetic cause of reported BOFS cases.

To date, 44 mutations in *TFAP2A* have been reported with BOFS according to the HGMD Professional 2019.4 and literature in Pubmed (Table S5). It includes 29 missense mutations, one nonsense mutation, one splicing mutation, two regulatory mutations, five small deletions/insertions, one small indel, five gross deletions. The disease-causing mutational hotspot region is in highly conserved DNA-binding domain of AP-2a, encoded by the exons 4 and 5 of *TFAP2A* gene with missense mutations in 90% cases (10).

*TFAP2A* mutations are associated with BOFS. Like most dominant diseases, BOFS expresses a considerable phenotypic variabilities. The patients displayed variable severity of clinical symptoms, even with the same mutation in the inter-intrafamily due to incomplete penetrance (5, 15–17) and somatic mosaicism (10). Titheradge et al. (18) reported a three generational BOFS family with c.703G >A, p.(Glu235Lys), demonstrating wide phenotypic spectrum, including lethality. In addition, modifier genes and/or enhancer genes were described to influence the clinical variability (3, 9).

No clear genotype-phenotype correlation has been clarified, however, there is a phenomenon that missense mutations appear with more severe features than the patients with the complete deletion of *TFAP2A* allele. It ascribes that the mutant proteins cause a dominant-negative activity on the wild-type AP-2a protein (19, 20).

The mutation c.912C>A, p.(Cys304^*^) in the present report is on exon 6, corresponding to HSH domain. A mutation in this domain may influence the ability of DNA binding and hinders the role of transcription.

I-1 and II-1 had mild symptoms, presented with isolated choroid coloboma, without systemic abnormalities. We speculated that this PTC mutation decreased mRNA levels through the mechanism of NMD. Therefore, the clinical features of the patients were milder than that of the patients with missense mutations. Further investigations need to explain why the manifestations are so mild in the patients.

*TFAP2A* is a retinoic acid (RA)-responsive gene to direct ocular morphogenesis. *Tfap2a* mutants in mice and zebrafish revealed a variable spectrum of eye phenotypes. *TFAP2A* gene regulates lens development and optic fissure closure (9, 21). Almost 83% of published BOFS cases involved the ocular anomaly (15). Individuals with *TFAP2A* mutations can present predominantly ocular phenotypes in the absence of branchial and craniofacial defects. The BOFS cases with predominantly ocular phenotypes were also listed in Table S5. Dumitrescu et al. (22) described a family harbored a heterozygous c.1150C>T, p.(His384Tyr) mutation in *TFAP2A* with primarily ocular involvement. Ng et al. (8) reported a family (affected child and mother) with a heterozygous c.253dupC, p.(Leu85Profs^*^84) mutation in *TFAP2A* gene presented with predominantly ocular anomalies. *TFAP2A* gene plays a role in the morphogenesis of the eye in animal models (23, 24). Ocular abnormalities of *TFAP2A* mutations are also variably expressed. Our two patients had isolated choroidal coloboma and did not have distinct non-ocular phenotypes, which was extremely rare in reported BOFS patients.

There are many hereditary diseases accompanied by ocular anomalies. Especially for the incomplete clinical appearance of the syndrome, they are at risk of being under-diagnosed and inadequately managed. Genetic analysis with NGS panel targeting more comprehensive ocular anomalies related genes helps to facilitate proper clinical diagnosis and improve the efficacy of genetic counseling for these disease groups.

To our knowledge, only one nonsense mutation c.740C>A, p.(Ser247^*^) in exon 4 (B domain) of *TFAP2A* gene in an affected daughter and mother pair was reported in available literature (16). In our report, the heterozygous mutation c.912C>A, p.(Cys304^*^) was the second nonsense mutation described to date, which caused the BOFS.

In summary, we applied NGS panel to a patient with bilateral choroidal coloboma who would not be led to a diagnosis of BOFS previously. We identified a novel nonsense mutation in *TFAP2A* in a highly conserved region of the HSH domain, which may be the pathogenic factor of the family. The findings contributed to the understanding of the genotype-phenotype correlation of BOFS. It can offer more knowledge for clinicians to make a clinical diagnosis with incomplete clinical features of the BOFS.

NGS is a powerful method to identify the genetic cause and improve genetic counseling for less clarified hereditary ocular diseases.

## Data Availability Statement

The raw datasets generated of this study are deposited on the CNGB Nucleotide Sequence Archive (CNSA: https://db.cngb.org/cnsa; accession number CNP0000402). They are available from the corresponding author on a reasonable application.

## Ethics Statement

This study was approved by the BGI-Shenzhen Ethics Committee (BGI-IRB19097). The proband and her spouse provided written informed consent for participation in the present study. In addition, the couple signed their daughter's informed consent as guardians. This study was performed based on the Principles of the Declaration of Helsinki. The couple provided written informed consent of themselves and their daughter as guardians for information and images to be published.

## Author Contributions

JM and YW provided clinical data and samples from the patient and her family. BM and HW designed the research and wrote the first draft of the article. XH conducted molecular genetic experimental studies. JM, BM, and SG analyzed data. All authors contributed to the article and approved the submitted version.

## Conflict of Interest

The authors declare that the research was conducted in the absence of any commercial or financial relationships that could be construed as a potential conflict of interest.
