# Supplementary material for: A Heterozygous Novel Mutation in TFAP2A Gene Causes Atypical Branchio-Oculo-Facial Syndrome With Isolated Coloboma of Choroid: A Case Report
Source: Front Pediatr. 2020 Jul 17;8:380. doi: 10.3389/fped.2020.00380 (PMC7379893; doi:10.3389/fped.2020.00380)
Supplement: Supplementary file 4 [file Table_4.DOCX]

Table S4. Primer pairs designed for identification of the mutations by Sanger sequencing.

| Mutation site | Primers (5’-3’) | Amplicon (bp) | Melting temperature (℃) |
| --- | --- | --- | --- |
| TFAP2A (NM_001042425.2); c.912C>A p.(Cys304*) | F: TTGACAACGAGACACAGAGACC  R: CTGGAGGTTGGTGGATGAGATT | 438 | 58 |
| ADAM9 (NM_003816.2);  c.1130+2dup | F: ATTTATCACGTAGGCTGTCAAC  R: ACTTCTCATTGGTATGTCATGC | 404 | 55 |
| C1QTNF5 (NM_015645.5);  c.43G>A p.(Gly15Ser) | F: GCCTGCCTGTGCTCTCC  R: ACGGGTACCTCCTCCACC | 585 | 60 |
| EYS (NM_001142800);  c.2791G>A p.(Glu931Lys) | F: GCCAGGATTATGGTGACTGTGAA  R: ATCAGTGGTGATCAGCTGCCT | 398 | 61 |

F, forward; R, reverse
